# Gradient Infiltration of Neutrophil Extracellular Traps in Colon Cancer and Evidence for Their Involvement in Tumour Growth

**DOI:** 10.1371/journal.pone.0154484

**Published:** 2016-05-02

**Authors:** Stella Arelaki, Athanasios Arampatzioglou, Konstantinos Kambas, Charalampos Papagoras, Paraskevi Miltiades, Iliana Angelidou, Alexandros Mitsios, Ioannis Kotsianidis, Panagiotis Skendros, Efthimios Sivridis, Ioanna Maroulakou, Alexandra Giatromanolaki, Konstantinos Ritis

**Affiliations:** 1 Department of Pathology, University General Hospital of Alexandroupolis, Alexandroupolis, Greece; 2 Laboratory of Molecular Hematology, Democritus University of Thrace, Alexandroupolis, Greece; 3 Laboratory of Cancer Genetics, Department of Molecular Biology and Genetics, Democritus University of Thrace, Alexandroupolis, Greece; 4 Department of Hematology, Democritus University of Thrace, Alexandroupolis, Greece; The Hospital for Sick Children and The University of Toronto, CANADA

## Abstract

**Background:**

The role of neutrophils in tumour biology is largely unresolved. Recently, independent studies indicated either neutrophil extracellular traps (NETs) or Tissue Factor (TF) involvement in cancer biology and associated thrombosis. However, their individual or combined role in colonic adenocarcinoma is still unexplored.

**Methods:**

Colectomy tissue specimens and variable number of draining lymph nodes were obtained from ten patients with adenocarcinoma of the colon. NETs deposition and neutrophil presence as well as TF expression were examined by immunostaining. The effect of NETs on cancer cell growth was studied in *in vitro* co-cultures of Caco-2 cell line and acute myeloid leukemia primary cells. Proliferation and apoptosis/necrosis of cancer cells were analyzed by flow cytometry.

**Results:**

TF-bearing NETs and neutrophil localization were prominent in tumour sections and the respective metastatic lymph nodes. Interestingly, neutrophil infiltration and NETs concentration were gradually reduced from the tumour mass to the distal margin. The *in vitro*-generated NETs impeded growth of cancer cell cultures by inducing apoptosis and/or inhibiting proliferation.

**Conclusions:**

These data support further the role of neutrophils and NETs in cancer biology. We also suggest their involvement on cancer cell growth.

## Introduction

Cancer is a leading cause of morbidity and mortality worldwide, with metastasis and cancer-associated thrombosis comprising the main causes of death in patients with malignancy. It is well known that the relationship between cancer and the immune system is not limited only to surveillance against rising monoclonal disorders, but also against established malignant tumors. The interplay between the immune system and cancer determines many aspects of the clinical presentation and the progression of the disease [1]. However, although neutrophils generally constitute the most prominent inflammatory cell population, relatively little is known about their implication in human tumours. Recent studies demonstrated that neutrophils mediate the crosstalk between cancer and immune system [2,3]. Tumour-infiltrating neutrophils have also been reported to facilitate metastasis, mainly by altering the microenvironment of the metastatic lesion [4–6]. Moreover, neutrophilia has been associated with a poorer prognosis in cancer patients [7], while the neutrophil to lymphocyte ratio has been proposed as a prognostic factor in several types of cancer, such as colon cancer [8].

Recently, a key mechanism of neutrophils, Neutrophil Extracellular Traps (NETs), has redefined their role in tumour biology [4–6,9–11]. NETs are extracellular chromatin structures composed of cytoplasmic, granular and nuclear components of neutrophils, initially described as an antimicrobial response [12,13]. However, there is increasing evidence for the key role of NETs in non-infectious diseases, such as thrombosis [14,15], autoimmune [16], autoinflammatory [17], cardiovascular diseases [15], fibrosis [18] and cancer [11]. To date, it has been suggested that NETs may act within the primary tumour promoting tumour progression [4,11], while at remote sites they might sequester circulating cancer cells favoring metastasis [5,6,19]. Additionally, NETs have been implicated in cancer-associated thrombosis [9,11].

Moreover, Tissue factor (TF) the main *in vivo* initiator of coagulation has been reported to play a key role in cancer biology [20,21]). TF-thrombin axis, apart from its implication in cancer-associated thrombosis, has been indicated to have a very potent angiogenic property via signaling of its serine proteases through PARs. Thus, this pathway has been implicated in neoangiogenesis and cancer metastasis [22]. Among cells able to express TF, cancer cells and neutrophils constitute source of this procoagulant and proinflammatory agent. However, the combined role of TF and NETs in cancer biology has never been previously addressed.

In this study we investigated the presence of NETs in solid tumours and metastatic lymph nodes of colon adenocarcinoma patients and their *in vitro* effects in cultures of colon cancer cells and primary leukemic cells. We demonstrate for the first time that NETs are present in surgical specimens of colon adenocarcinoma and the respective metastatic lymph nodes and they are also decorated with TF. We also report that NETs may limit cancer cell growth *in vitro* by inducing apoptosis and/or inhibiting proliferation.

## Materials & Methods

### Human samples

For this study, formalin-fixed paraffin-embedded surgical tissue specimens from 10 patients who had undergone colectomy for adenocarcinoma, including the draining lymph nodes, were examined. Patient characteristics are demonstrated in S1 Table. Sections were taken in succession per one cm from the center of the tumour mass up to the surgical margin from every sample of colon adenocarcinoma. Moreover, both metastatic and non-metastatic indentified lymph nodes were examined.

In addition, 10 healthy donors were enrolled for blood samples in order to isolate polymorphonuclear neutrophils (PMNs).

Written consent was granted by all individuals involved in this study. The study protocol design was in accordance with the Declaration of Helsinki and was approved by the Ethics Review Board of the University Hospital of Alexandroupolis.

### Immunohistochemistry/Immunofluorescence

Sections were deparaffinized and immunocytochemical staining was performed using a LSAB/HRP kit (DAKO) as previously described [23]. Neutrophil elastase was detected with an IgG rabbit polyclonal anti-neutrophil elastase antibody (1/50 dilution; Santa Cruz, CA, USA; sc-25621). Sections were then counterstained with hematoxylin, dehydrated and mounted. Mouse monoclonal IgG1 was used as negative control. Samples were visualized under light microscopy (Nikon, model Eclipse E400) and images were captured using a Nikon Digital Camera (ACT-1 Nikon software).

For immunofluorescence, sections were stained as previously described [24]. Nonspecific binding sites were blocked with 2% goat serum in 2% BSA—PBS. NETs were stained with a rabbit anti–citrullinated histone H3 polyclonal antibody (Abcam, UK; ab5103), a mouse anti—NE monoclonal Antibody (Santa Cruz; sc-55548), a rabbit anti—NE poluclonal antibody (Santa Cruz; sc-25621) and a mouse anti—myeloperoxidase monoclonal antibody (Santa Cruz; sc-52707). For TF detection an IgG1—TF mAb (Sekisui Diagnostics, Lexington, USA; 4508) was used. A goat anti-rabbit Alexa fluor 647 antibody (Invitrogen, Carlsbad, USA; A21244) and a polyclonal rabbit anti-mouse Alexa fluor 488 antibody (Invitrogen; A11059) were utilized as secondary antibodies. Sections were counterstained with DAPI, mounted and visualized in a confocal microscopy (Spinning Disk Andor Revolution Confocal System, Ireland) in a PLAPON 606O/TIRFM-SP, NA 1.45 and UPLSAPO 100XO, NA 1.4 objectives (Olympus, Hamburg, Germany).

### Cell isolation

Peripheral blood neutrophils were isolated from heparinized blood from healthy donors as previously described [24]. Primary human acute myeloid leukemia (AML) cells were isolated using Biocoll Separating Solution according to the manufacturer's instructions (Biochrom, Berlin, DE) from peripheral blood samples derived from patients at the University Hospital of Alexandroupolis, Greece. AML diagnosis was made in accordance with the World Health Organization criteria.

### Cell culture, stimulation and inhibition studies

Caco-2 [Caco2] (ATCC^®^ HTB-37^™^) (ATCC, Manassas, USA) cells were cultured in 5% CO_2_, at 37°C, in L-glutamine Eagle's Minimum Essential Medium (EMEM; Gibco BRL, New York, USA). AML cells were cultured in 5% CO_2_, at 37°C, in Myeloid Long-Term Culture Medium (MyeloCult^™^ H5100; Stem Cell Technologies, Vancouver, Canada). Both Caco-2 and AML cells were stimulated with 500 ng NET structures isolated from neutrophils treated with the aforementioned agents, or whole PMN pretreated with these agents, for 4 days. For NET scaffold inhibition, isolated NET structures were pre-incubated with DNase1 (10 U/ml; Fermentas, Vilnius, Lithuania) or heparin (heparin sodium, 100 μg/ml; LEO Pharma A/S, Ballerup, Denmark) for 60 min. For TF inhibition an IgG1 mouse anti-human TF mAb (10 μg/ml; Sekisui Diagnostics) was used.

Four images randomly taken from different regions of each well with 100x magnification per experiment were analysed. Average surface area covered by cells was calculated with Fiji/ImageJ [25].

### NET structure generation, isolation and quantification

To generate NETs, 1.5 × 10^6^ neutrophils were seeded in six-well culture plates (Corning Incorporated, New York, USA) in low-serum RPMI medium (Gibco BRL) as previously described [13] and were treated with sepsis serum [14], isolated from blood samples derived from septic patients at the Academic Hospital of Alexandroupolis, Greece, for 210 min. Neutrophils were also incubated with phorbol 12-myristate 13-acetate (PMA) (40 ng/ml; Sigma-Aldrich, St Louis, MO, USA), a generic inducer of NET release, for 210 min. Afterwards, the medium was removed and cells were washed with RPMI. Moreover, 1 ml RPMI was added to each well and NETs were collected after vigorous agitation. The medium was centrifuged at 20xg for 5 min and NETs were collected in supernatant phase. These concentrations and time points were optimal for neutrophil stimulation according to optimizing experiments. DNA content was determined using Nanodrop.

### Cell proliferation and apoptosis

For the analysis of cell proliferation, Caco-2 and AML cells were stained with 5(6)-carboxyfluorescein diacetate N-succinimidyl ester (CFSE; Sigma-Aldrich) [26], CD34 APC (clone 8G12; BD Biosciences, New Jersey, USA) and CD45 PerCp (clone 2D1; BD Biosciences) before stimulation. Experiments were performed and analyzed in timer series experiments considering as paternal population for comparison the cells with highest CFSE intensity, according to landmark publications [27,28].

For the analysis of apoptosis/necrosis, Caco-2 and AML cells were stained with FITC-annexin V (BD Biosciences) and propidium iodide (PI; Sigma-Aldrich). AML cells were also stained with CD34 APC (clone 8G12; BD Biosciences).

Proliferation and apoptosis analysis were performed after 4 days of co-culture with NETs in a FACScalibur flow cytometer (BD Biosciences). All data were analyzed with Flowjo V10.

### Statistical analysis

Statistical analyses were performed using one-way analysis of variance (ANOVA) with Scheffé test for post hoc comparisons. P values less than 0.05 were considered significant. All statistical analyses were performed with OriginPro 8.

## Results

### NETs are present in human colon adenocarcinoma and metastatic lymph nodes

Since it has been recently suggested that NETs are implicated in cancer progression and metastasis in murine lung and mammary tumour models [4,11], we investigated whether they are present in human colon adenocarcinoma and their respective metastatic lymph nodes.

**Fig 1:**
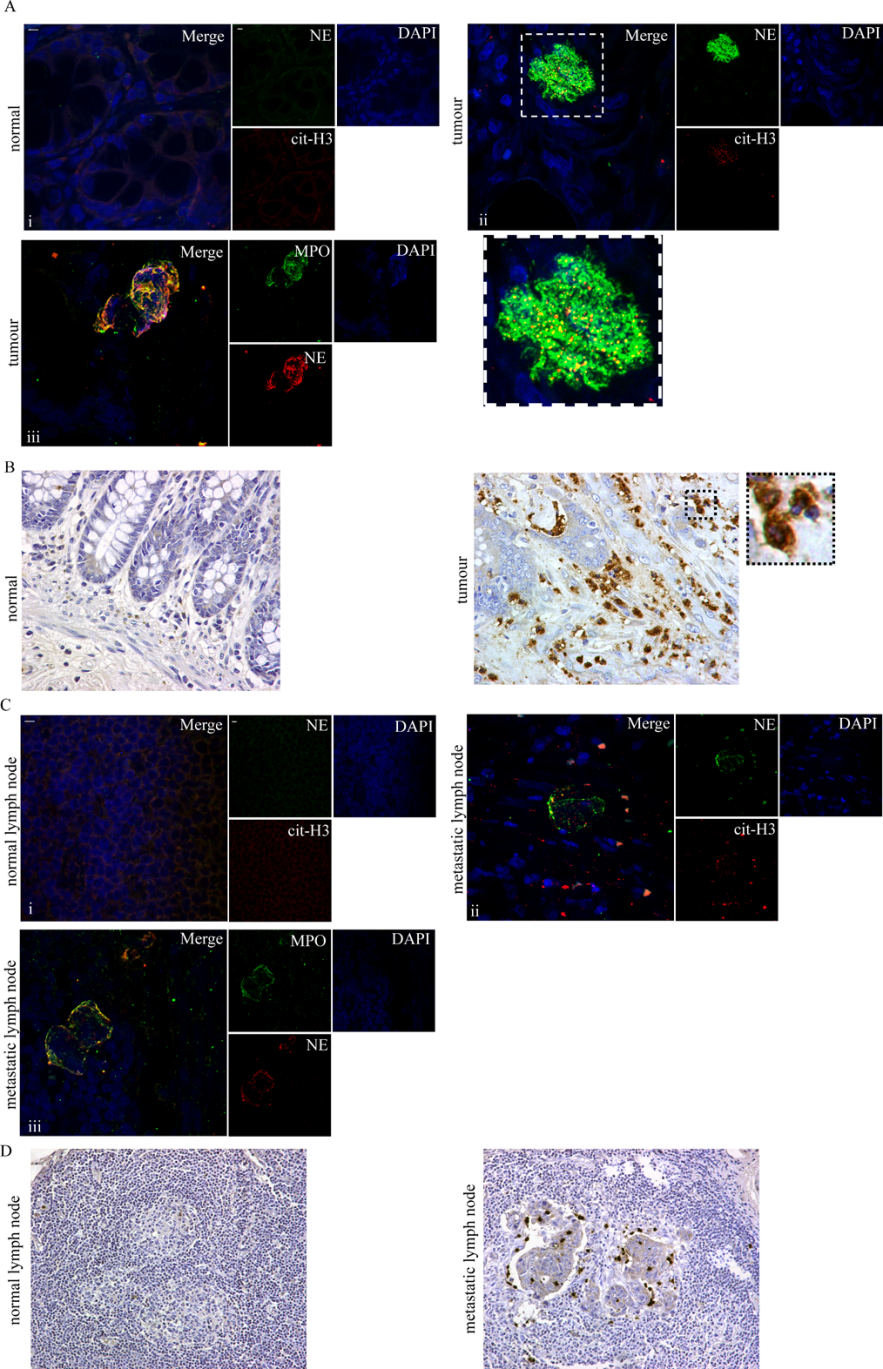
Presence of NETs and neutrophils in colon adenocarcinoma tumour and metastatic lymph nodes. (A) NETs visualized in colon adenocarcinoma specimens as extracellular structures decorated with neutrophil elastase and citrullinated H3 or neutrophil elastase and MPO, (confocal microscopy). (B) Abundance of neutrophil presence in colon adenocarcinoma demonstrated by neutrophil elastase immunohistochemical staining (light microscopy). Magnified section demonstrates neutrophils stained with neutrophil elastase. NETs (C) and neutrophil (D) presence in metastatic lymph nodes in patients with colon adenocarcinoma. (C) NE/cit-H3 and NE/MPO immunofluorescence confocal microscopy and (D) neutrophil elastase immunohistochemical staining. (A), (C) i-ii Green: NE, Red: cit-H3, Blue: DAPI/DNA, iii Green: MPO, Red: NE, Blue: DAPI/DNA (A), (B), (C), (D) One representative out of ten independent experiments is shown. (A), (C) Original magnification 600x, Scale bar– 5μm. Original magnification (B) 400x, (D) 200x.

To this end, we used confocal microscopy to examine tumour specimens of colectomy for adenocarcinoma from ten patients. We observed significant deposition of NETs in tumour sections, as extracellular colocalization of neutrophil elastase with citrullinated H3 (Fig 1Ai and 1Aii) and neutrophil elastase with myeloperoxidase (Fig 1Aiii) in confocal microscopy, in contrast to their absence in normal intestine sections of the same patients. In particular, NETs were localized in the proximity of cancer cells and in the stroma (Fig 1Aii) and even within glandular lumens of the main colonic mass. To verify our observation in lower magnification, the same tissue samples were examined for neutrophil presence by neutrophil elastase immunohistochemistry. Neutrophils stained with NE demonstrated similar localization in the tumour as NETs (Fig 1B). Expectedly, neutrophils were also visible with haematoxylin and eosin (H&E) staining using higher magnification (400x) (S1 Fig), although they were more readily visible by the immunohistochemical method.

Furthermore, NETs (Fig 1C) and neutrophils (Fig 1D) were also detected in sections of the respective metastatic lymph nodes, while they were absent in the remaining normal lymph nodes of the same patient.

Together, these data indicate that both neutrophils and NETs are present in the vicinity of cancer cells.

### Neutrophils and NETs are reduced proportionally to the distance from the tumour mass and they are a prominent source of TF in cancer microenvironment

Considering the presence of inflammation in cancer and that inflammation generates a concentration gradient of cytokines and chemokines [29], we next investigated whether NETs follow a similar pattern of concentration gradient from the core of the tumour towards the neighboring healthy tissue.

**Fig 2:**
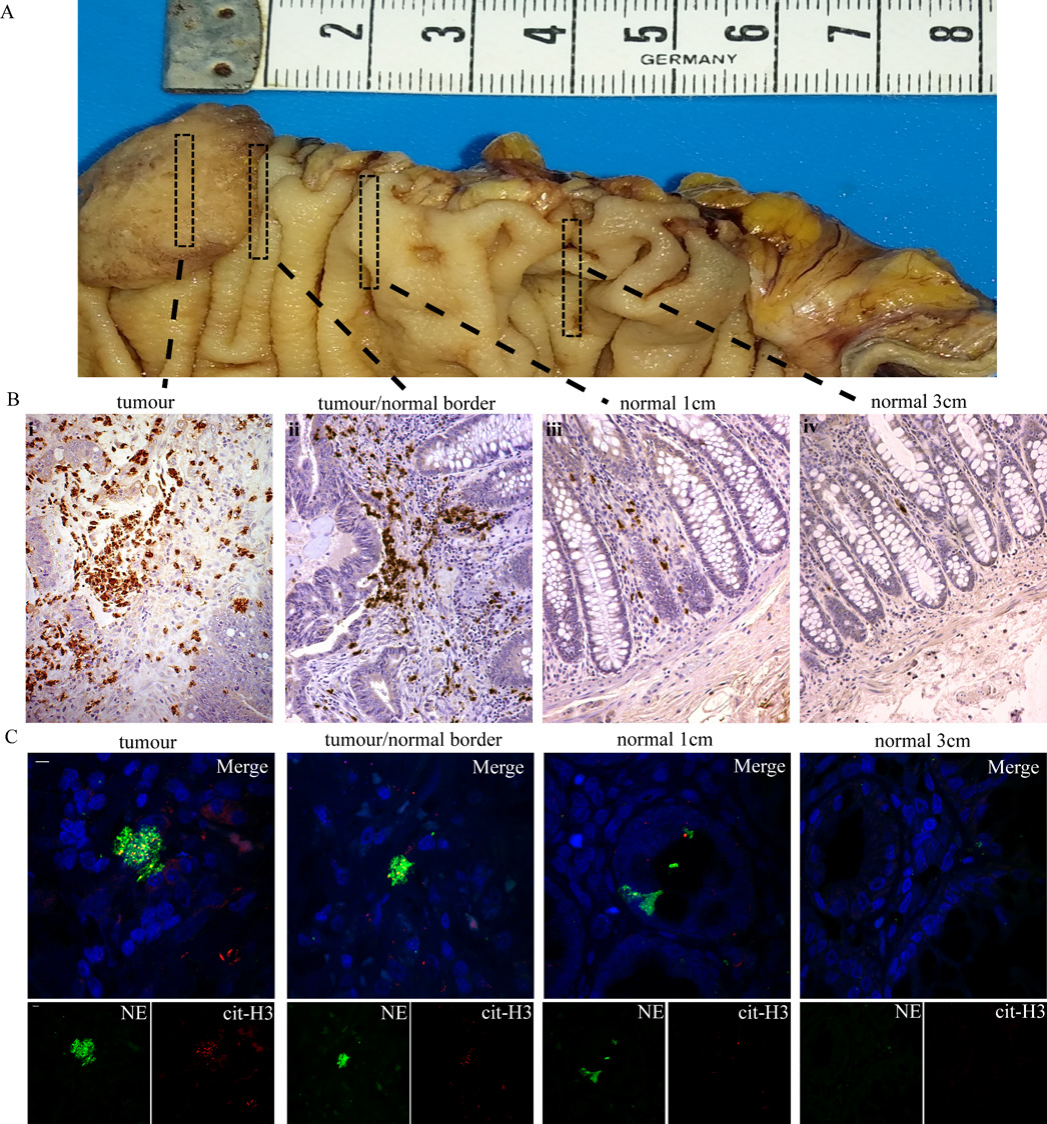
Neutrophil and NETs presence is diminished accordingly to the distance from the center of tumour. —Representative experiment where NETs and neutrophils were diminished at 3 cm from tumour mass. (A) Macroscopic photography of colectomy biopsy specimen for demonstration of successive section sampling. (B) Neutrophil elastase immunohistochemical staining in successive biopsy specimens from the center of tumour, up to 3–4 cm from the tumor mass in healthy tissue in patients with colon adenocarcinoma. (C) Neutrophil elastase and citrullinated H3 immunofluorescence staining in successive biopsy specimens from the center of tumour, up to 3–4 cm from the tumor mass in healthy tissue in patients with colon adenocarcinoma. Green: NE, Red: cit-H3, Blue: DAPI/DNA. (B),(C) One representative out of ten independent experiments is shown. Original magnification (B) 200x, (C) 600x, Scale bar– 5μm.

We observed a gradient of neutrophil accumulation in proportion to the distance from the center of the tumour mass (Fig 2A), determined by neutrophil elastase immunohistochemistry (Fig 2B). Interestingly, a small presence of neutrophils was still observed in sections of normal colon immediately adjacent to the tumour and gradually declined in parallel to the distance up to the point of complete absence (Fig 2B, iv). For further details on the distance see S1 Table. As expected, NET concentration also declined similarly to neutrophils, as assessed by confocal microscopy (Fig 2C).

**Fig 3:**
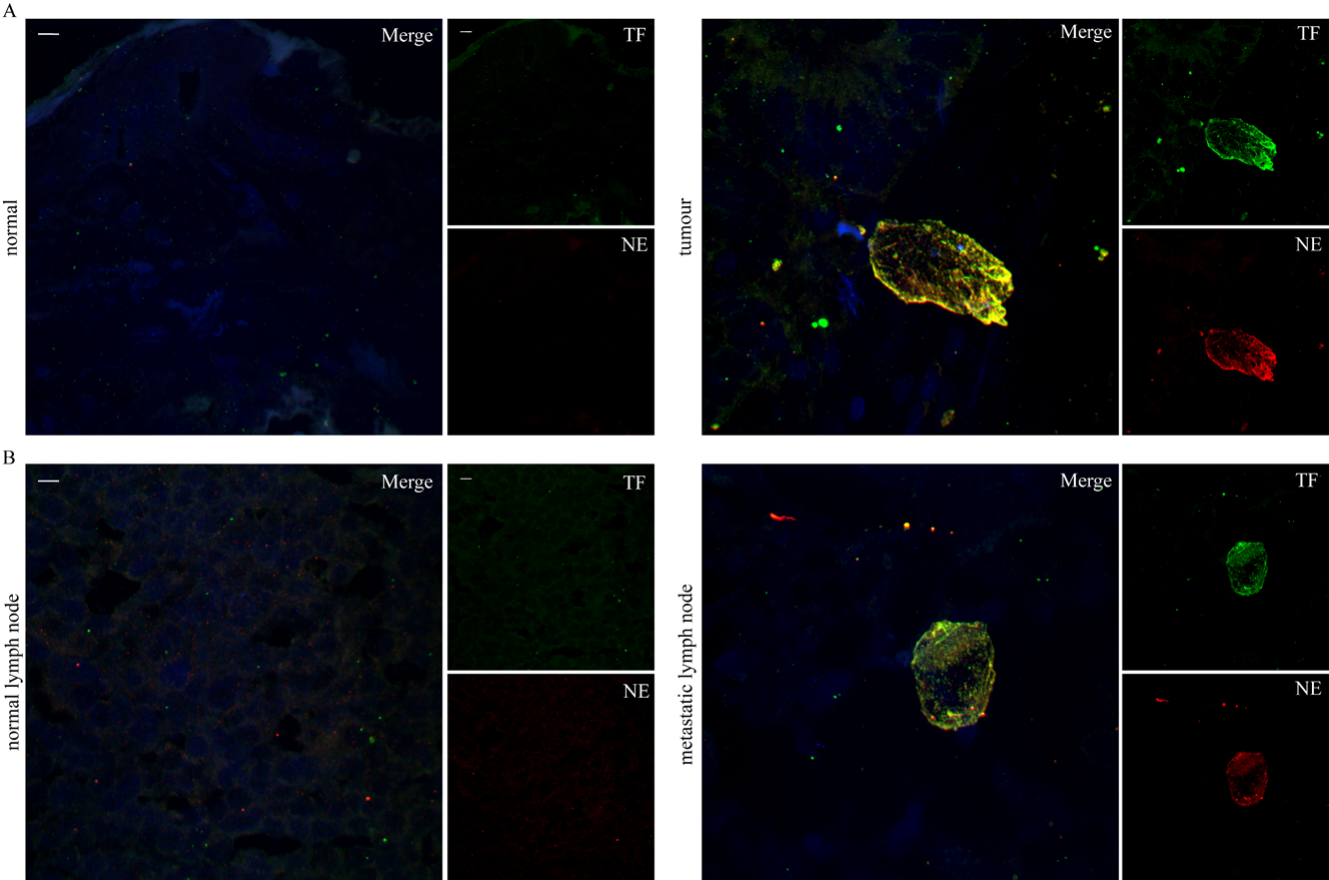
TF localization on NETs present in colonic adenocarcinoma and metastatic lymph nodes. TF and neutrophil elastase immunofluorescence staining in (A) colonic adenocarcinoma specimens and (B) respective metastatic lymph nodes. Green: TF, Red: NE, Blue: DAPI/DNA. One representative out of four independent experiments is shown. Original magnification 600x, Scale bar– 5μm.

These findings indicate that the intensity of neutrophil infiltration and NET generation is proportional to the proximity to the tumour.Since TF has a key role in tumour biology and thromboinflammation and recent publications demonstrate that, in thrombotic disorders, NETs express functional TF [14,15,16], we examined the presence of TF on NETs localized within the tumour mass and the metastatic lymph nodes. Indeed, TF was detected mainly on NETs as observed by colocalization with extracellular neutrophil elastase in confocal microscopy (Fig 3A and 3B).

These data suggest that neutrophils and NETs are important sources of TF in tumour microenvironment.

### NET structures inhibit *in vitro* growth and induce apoptosis in colon cancer cells

Based on the above findings we investigated the possible role of NETs in solid tumour progression in *in vitro* co-cultures of Caco-2 cells, a colon cancer cell line. In addition, to further examine the role of TF, TF-negative (PMA-induced) and TF-bearing (sepsis serum-induced) NETs were used [14].

**Fig 4:**
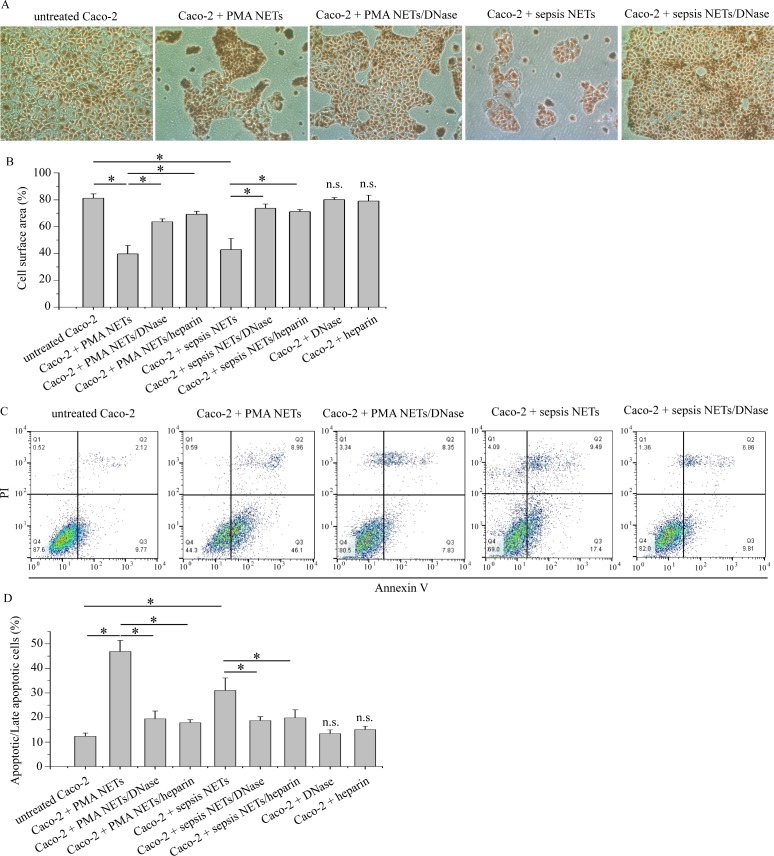
NETs inhibit growth and induce apoptosis in Caco-2 cultures. A) May Grünwald-Giemsa staining of Caco-2 cells co-cultured with PMA or sepsis serum-induced NETs in the presence or absence of NET scaffold inhibitors. One representative out of four independent experiments is shown. Original Magnification 100x. (B) Percentage of surface area covered by cells. (C), (D) Annexin V/PI flow cytometry of Caco-2 cells co-cultured with PMA or sepsis serum-induced NETs in the presence or absence of NET scaffold inhibitors. (C) Representative scatter plots. (B), (D) Data from four independent experiments presented as mean±SD. n.s.—not significant compared to control, *p < 0.05.

Both PMA and sepsis serum-induced NETs inhibited *in vitro* Caco-2 growth compared to controls (Fig 4A and 4B), which also demonstrated apoptotic-like morphology. Dismantling of NETs with DNase I or heparin abolished their inhibitory effect. Similar results were also obtained when either PMA or sepsis serum-pretreated neutrophils were used in Caco-2 cultures (S2A Fig).

We next investigated whether NETs inhibit Caco-2 growth via apoptosis. Thus, Caco-2 cells were treated with the aforementioned agents and demonstrated increased levels of apoptotic and late apoptotic cells, as assessed by Annexin V/PI flow cytometry (Fig 4C and 4D). DNase I or heparin attenuated this effect on apoptosis (Fig 4C and 4D). Moreover, apoptosis induction by NETs was concentration-dependent (data not shown).These data indicate that NETs, irrespectively of the NET-generating stimulus, act as potent inhibitors of colon cancer cells growth *in vitro* by inducing apoptosis in these cells.

### NETs also inhibit acute myeloid leukemia cells growth *in vitro*

To further clarify whether this inhibitory role of NETs in cancer cell growth is specific only to colon adenocarcinoma or occurs with other types of malignancy as well, we investigated their effects in human hematopoietic cancer. Thus, we conducted *in vitro* co-culture experiments of primary AML cells in the presence of NETs.

**Fig 5:**
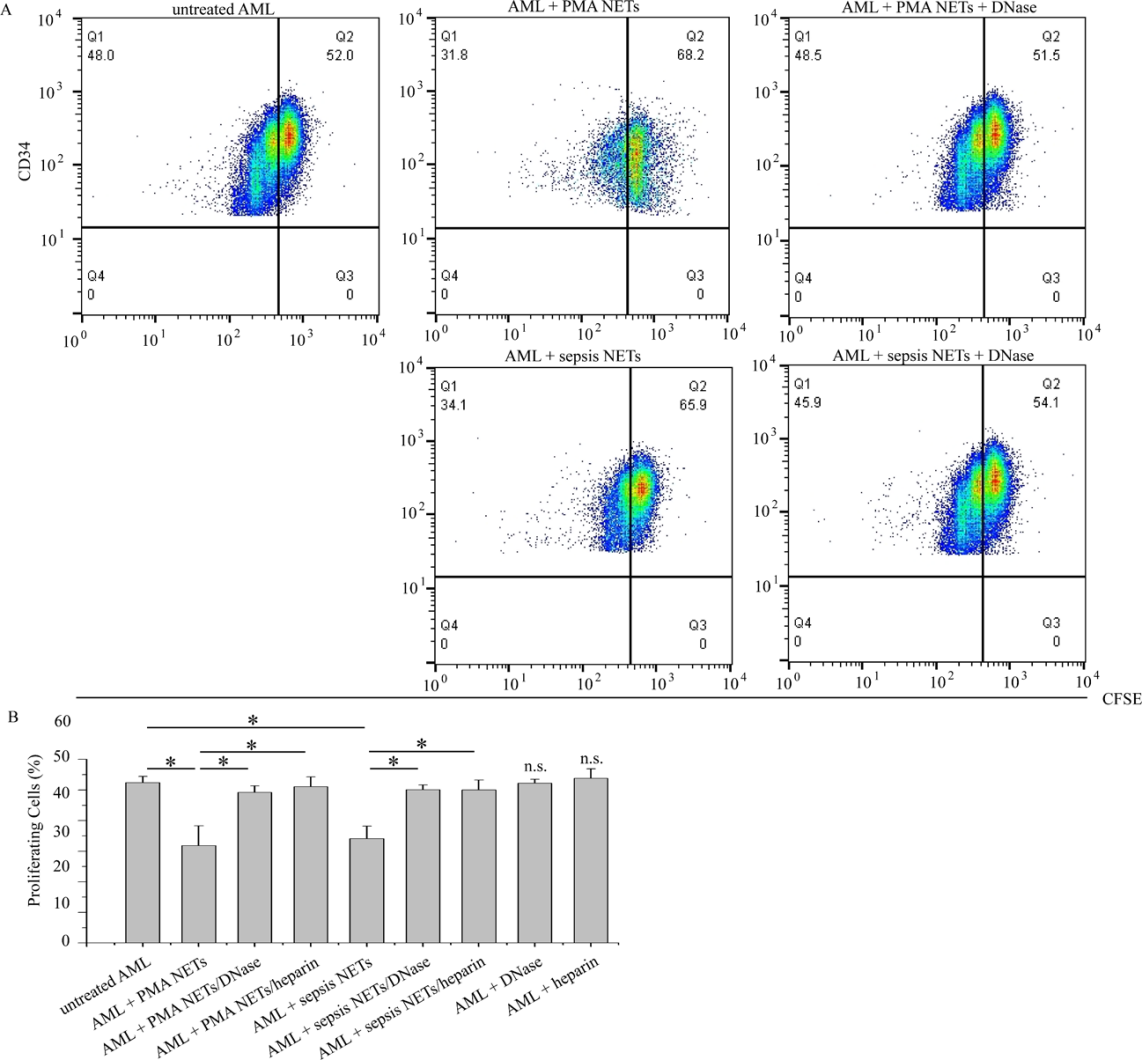
NETs reduce proliferation of AML cells. (A), (B) CFSE flow cytometry of AML cells co-cultured with either PMA or sepsis serum-induced NETs in the presence or absence of NET scaffold inhibitors. (A) Representative scatter plots. (B) Data from four independent experiments presented as mean±SD. n.s.—not significant compared to control, *p < 0.05.

Similarly to Caco-2 cells, PMA or sepsis-induced NETs or respectively pre-stimulated neutrophils significantly suppressed the growth of AML cells (S2B Fig) and induced apoptosis (S2C and S2D Fig). Additionally, NET-treated AML cells demonstrated a remarkable reduction of their proliferation compared to controls, as assessed by flow cytometry (Fig 5A and 5B). On the other hand, NET chromatin scaffold inhibitors (DNase or heparin) abolished NETs effects on both AML cell proliferation and apoptosis (Fig 5A and 5B, S2D Fig).

These data taken together with the previous findings suggest that NETs, irrespectively of the stimulus, have also a potentially universal inhibitory role in primary human cancer cell cultures, as was observed in cells of acute myeloid leukemia.

## Discussion

In this study, we demonstrated for the first time the presence of NETs, decorated with TF, in colon adenocarcinoma sections of both the primary tumour mass and the respective metastatic lymph nodes. We also provide evidence for a potential role of NETs to limit *in vitro* cancer cell growth by inducing apoptosis both in cultures of Caco-2 and primary AML cells. Moreover, NETs inhibited *in vitro* proliferation in AML cells.

In line with previous reports indicating the presence of NETs in cancer biopsies [6,19], we showed abundance of NETs in colon adenocarcinoma sections both within the primary tumour and in metastatic lymph nodes, as extracellular colocalizations of neutrophil elastase with citrullinated histone H3 or MPO. Moreover, we observed increased infiltration of neutrophils in these sections elucidating the source of these NETs. Notably, NET-generating neutrophils demonstrated an aggregated pattern, possibly as a result of neutrophils attaching on already formed NETs and further generating more NETs. Although increased presence of neutrophils was also observed with the standard histochemical H&E staining at high magnifications, immunohistochemical methods were superior for the identification of infiltrating neutrophils in the tissue. Additionally, we observed a gradual reduction of neutrophil infiltration and NET concentration from the core of the tumour towards the adjacent tissues, while, at more distal sections into the uninvolved colon, neutrophils and NETs were completely absent. Apparently, this reflects a gradient of inflammation emanating from the neoplastic lesion and spreading into the surrounding tissues. Since immunohistochemical staining provides more visible results concerning neutrophil infiltration and coupled with the absence of NETs and neutrophils at a certain distance from the tumour, this simple method could help to determine a proper surgical resection along with the standard pathological examination of the surgical excision margins.

We observed that NETs both in the primary tumour mass and in the metastatic lymph nodes are decorated with TF, the main *in vivo* initiator of coagulation and also an important angiogenic factor. TF has been shown by our group to occur in functional form on NETs [14,15]. This multifaceted role of TF taken together with its presence on NETs within primary and metastatic malignant lesions may have significant implications for tumour biology both locally and systemically. Locally, TF expression within the tumour may be a key to intra-tumour thrombosis and necrosis often observed in biopsies. On the other hand, TF may sustain local tumour growth by promoting neoangiogenesis which is essential for the metabolic support of the highly active malignant cells. On a systemic level, tumour-associated NETs may be a significant source of active TF which can alter the prothrombotic/antithrombotic balance of the body in favor of thrombosis. Indeed, thrombosis is one of the leading causes of death in cancer patients and unanticipated thrombosis is occasionally an early manifestation of an as yet undiagnosed cancer [30,31].

The biological significance of NETs present, however, remains unclear. On one hand, they may represent a reaction of the tumour environment against the growing cancer. On the other hand, they may play an adverse role in tumour growth offering a scaffold with an array of biologically active molecules attached on it, which may promote malignant cell survival, growth and, ultimately, local tumour expansion [4,11]. In our experiments, both sepsis-induced NETs which are able to express TF and PMA-induced “generic” NETs not containing TF, demonstrated similar inhibitory activity in cultures of a colon carcinoma cell line and primary human leukemic cells. More specifically, NETs induced apoptosis in both types of cancer cells and they reduced the proliferation of primary AML cells. This effect was NET-specific and dependent on the integrity of NET chromatin scaffold, since dismantling of NETs with different agents (DNase, heparin) abrogated those effects. Application of CFSE protocol on Caco-2 cells for determination of proliferation did not produce readable results since it does not perform properly on non-primary cell lines. These data suggest that NETs, independently of their triggering stimulus and the presence of TF, act as generic potent inhibitors of cancer cells by inducing apoptosis, while also reducing the proliferation rate. Considering a previous report demonstrating that NETs induce proliferation in primary human lung fibroblasts [18] and not apoptosis, further investigation is required in order to verify the specificity of the inhibitory effect of NETs on cancer cells. Our *in vitro* findings are not in accordance with recent reports that demonstrate a pro-tumorigenic activity of NETs [6,9,19]. Thus, we could propose that NETs may have two opposing faces in cancer environment. The first one indicates the effort of NETs, as an initial inflammatory response, to restrict the tumor. The second face may reflect their adjustment in the neoplastic microenvironment and “collaboration” with tumor cells. Considering that different inflammatory conditions generate NETs decorated with different components [15,17,18,32], such as TF we could explain why NETs might have a two-fold action in cancer, either anti-tumorigenic or pro-metastatic. Whether they act towards either way, it is possibly a matter of tumour microenvironment, inflammatory response and NET-bound components.

In conclusion, we show here for the first time the presence of NETs in colon cancer tissue and the respective metastatic lymph nodes. The pattern of neutrophilic distribution close to the area of tumour and neutrophil specific immunohistochemical method could provide a useful tool for the determination of proper surgical margin. The TF immunostaining is more prominent on NETs than in cancer cells pointing neutrophils as a significant source of TF in cancer microenvironment. Moreover, the subsequent thromboinflammatory and angiogenetic activity of TF may be involved in thrombosis and metastasis. Although NETs in our *in vitro* experiments suppress the growth of cancer cells, further studies are required to elucidate the mechanisms by which neutrophils are involved in cancer growth. These findings on this largely neglected cellular population in cancer provide a springboard for further investigation and indicate neutrophils and NETs as possible therapeutic targets.

## Supporting Information

S1 Fig Presence of neutrophils in colon cancer and metastatic lymph nodes. H&E staining of colon adenocarcinoma patient specimens and respective metastatic lymph node specimens. Arrows demonstrate neutrophils. One representative out of ten independent experiments is shown. Original magnification 400x.
(TIF)

S2 Fig NETs inhibit growth of cancer cells and induce apoptosis. (A) May Grünwald-Giemsa staining of Caco-2 cells co-cultured with PMNs pretreated with PMA or sepsis serum. One representative out of four independent experiments is shown. Original Magnification 100x. (B) May Grünwald-Giemsa staining of AML cells co-cultured with either PMA or sepsis pretreated PMNs, or PMA or sepsis serum-induced NETs in the presence or absence of NET scaffold inhibitors. One representative out of four independent experiments is shown. Original Magnification 100x. (C) and (D) Annexin V/PI flow cytometry of AML cells co-cultured with PMA or sepsis serum-induced NETs in the presence or absence of NET scaffold inhibitors. (C) demonstrates representative scatter plots. (D) Data from four independent experiments presented as mean±SD. n.s.—not significant compared to control, *p < 0.05.
(TIF)

S1 Table Clinical Characteristics of Patients with Colorectal Cancer. (DOC)
