# Supplementary figures and images for: Gradient Infiltration of Neutrophil Extracellular Traps in Colon Cancer and Evidence for Their Involvement in Tumour Growth
Source: PLoS One. 2016 May 2;11(5):e0154484. doi: 10.1371/journal.pone.0154484 (PMC4852909; doi:10.1371/journal.pone.0154484)

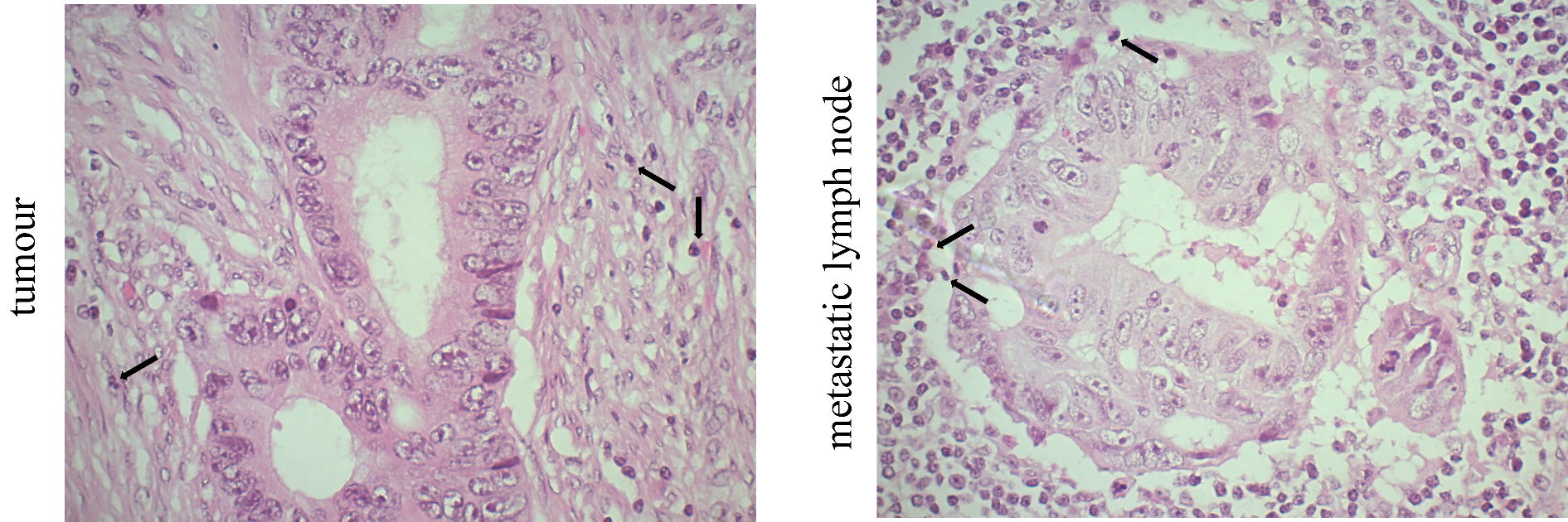

Supplement: S1 Fig — H&E staining of colon adenocarcinoma patient specimens and respective metastatic lymph node specimens. Arrows demonstrate neutrophils. One representative out of ten independent experiments is shown. Original magnification 400x. (TIF) [file pone.0154484.s001.tif]

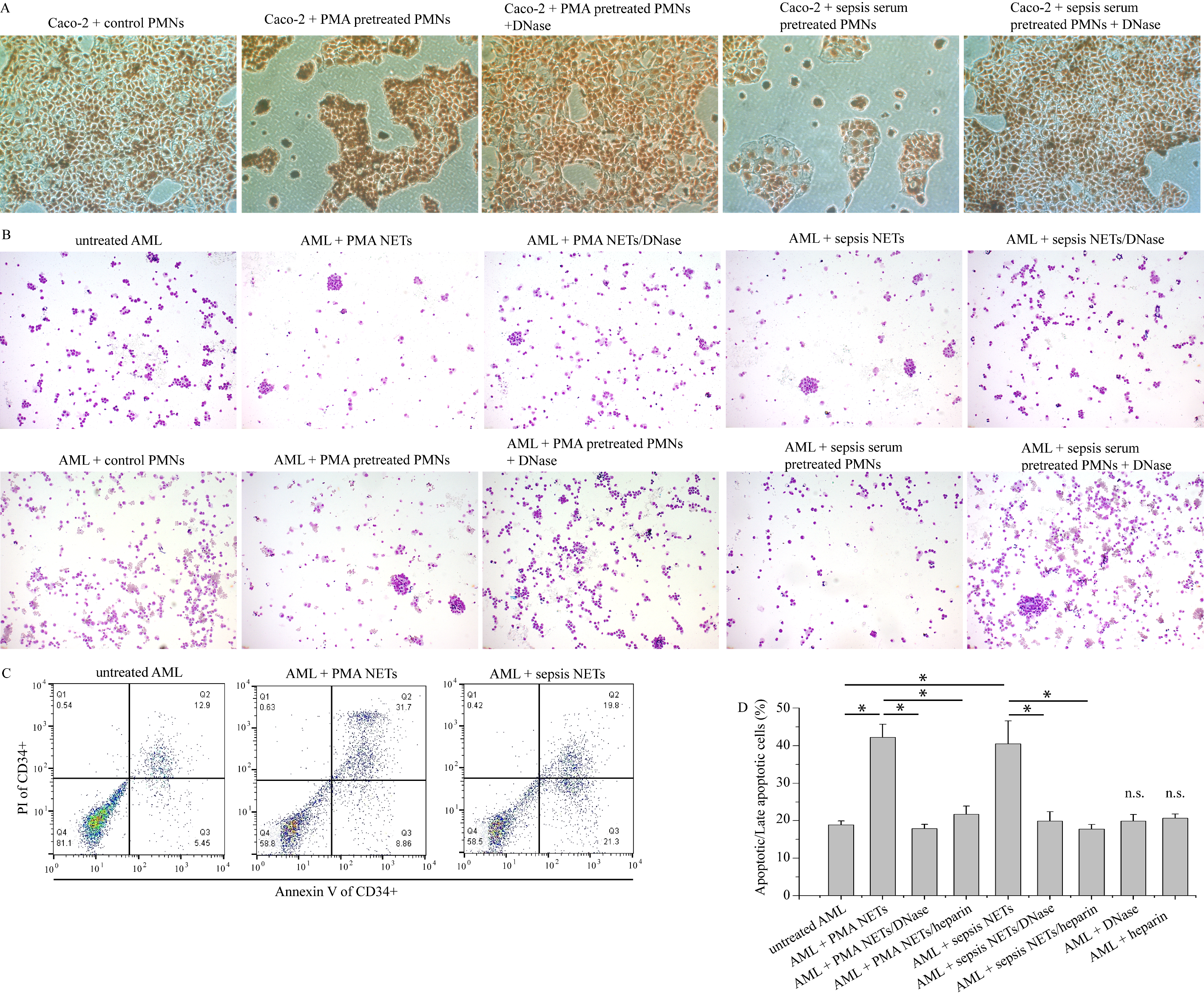

Supplement: S2 Fig — (A) May Grünwald-Giemsa staining of Caco-2 cells co-cultured with PMNs pretreated with PMA or sepsis serum. One representative out of four independent experiments is shown. Original Magnification 100x. (B) May Grünwald-Giemsa staining of AML cells co-cultured with either PMA or sepsis pretreated PMNs, or PMA or sepsis serum-induced NETs in the presence or absence of NET scaffold inhibitors. One representative out of four independent experiments is shown. Original Magnification 100x. (C) and (D) Annexin V/PI flow cytometry of AML cells co-cultured with PMA or sepsis serum-induced NETs in the presence or absence of NET scaffold inhibitors. (C) demonstrates representative scatter plots. (D) Data from four independent experiments presented as mean±SD. n.s.—not significant compared to control, *p < 0.05. (TIF) [file pone.0154484.s002.tif]
