# Supplementary material for: Gradient Infiltration of Neutrophil Extracellular Traps in Colon Cancer and Evidence for Their Involvement in Tumour Growth
Source: PLoS One. 2016 May 2;11(5):e0154484. doi: 10.1371/journal.pone.0154484 (PMC4852909; doi:10.1371/journal.pone.0154484)
Supplement: S1 Table — (DOC) [file pone.0154484.s003.doc]

**Supporting Information**

| **S1 Table.**  **Clinical Characteristics of Patients^*^ with Colorectal Cancer** | | | | | | |
| --- | --- | --- | --- | --- | --- | --- |
|  | **Age  (yrs)** | **Gender  (M/F)** | **Staging^**^** | **Localization** | **Lymph nodes  (removed/**  **metastatic)^***^** | **Distance  (cm)^****^** |
| Pt. 1 | 83 | M | IIIC / T_3_N_2_M_0_ | Rectosigmoid | 16 / 11 | 4 |
| Pt. 2 | 62 | M | IIIB / T_3_N_1_M_0_ | Rectosigmoid | 11 / 2 | 3 |
| Pt. 3 | 82 | F | IIA / T_3_N_0_M_0_ | Rectosigmoid | 12 / 0 | 3 |
| Pt. 4 | 79 | M | IIIB / T_3_N_1_M_0_ | Ascending colon | 6 / 1 | 4 |
| Pt. 5 | 70 | F | IIA / T_3_N_0_M_0_ | Ascending colon | 12 / 0 | 3 |
| Pt. 6 | 67 | F | IIA / T_3_N_0_M_0_ | Cecum | 9 / 0 | 4 |
| Pt. 7 | 75 | M | IIIB / T_3_N_1_M_0_ | Descending colon | 13 / 1 | 4 |
| Pt. 8 | 78 | M | I / T_2_N_0_M_0_ | Rectosigmoid | 6 / 0 | 2 |
| Pt. 9 | 78 | M | IIA / T_3_N_0_M_0_ | Ascending colon | 13 / 0 | 3 |
| Pt. 10 | 62 | M | IIIB / T_3_N_1_M_0_ | Ascending colon | 13 / 1 | 4 |
| **Mean ±SD** | 73.6 ± 7.85 |  |  |  |  |  |

^*^ No patient received chemotherapy, or radiotherapy, or was suffering from another inflammatory, autoimmune or infectious disease.

^**^ Staging according to the American Joint Committee on Cancer (AJCC) and TNM. T: denotes the degree of invasion of the intestinal wall. N: denotes the degree of lymphatic node involvement. M: denotes the degree of metastasis.

^***^ Only metastatic lymph nodes (all) were infiltrated by neutrophils/NETs.

^****^ The distance in cm from the tumour mass where neither NETs deposition nor neutrophil infiltration were detected.
